# Self-administered succus entericus reinfusion before ileostomy closure improves short-term outcomes

**DOI:** 10.1186/s12893-021-01444-4

**Published:** 2021-12-28

**Authors:** Zhen Liu, Liang Fang, Liang Lv, Zhaojian Niu, Litao Hou, Dong Chen, Yanbing Zhou, Dong Guo

**Affiliations:** 1 Department of Emergency Surgery, The Affiliated Hospital of Qingdao University, Qingdao, Shandong China; 2 Department of Gastrointestinal Surgery, The Affiliated Hospital of Qingdao University, No.16 Jiangsu Rd, Qingdao, 266000 Shandong China; 3 Department of Gastroenterology, The Affiliated Hospital of Qingdao University, Qingdao, Shandong China; 4 Department of General Surgery, The Affiliated Hospital of Qingdao University, Qingdao, Shandong China

**Keywords:** Rectal cancer ileostomy closure, Succus entericus reinfusion, Clinical outcomes, Quality of life

## Abstract

**Objective:**

The study aims to assess whether reinfusion of succus entericus prior to ileostomy closure can decrease postoperative length of stay and ameliorate low anterior resection score.

**Methods:**

This study is a retrospective analysis based on prospectively collected data. Patients were screened from May 2016 to November 2019. A total of 30 patients who underwent reinfusion with succus entericus (SER) were enrolled in the SER group and 42 patients without SER were enrolled in the non-SER group.

**Results:**

There was no significant difference in the incidence of postoperative ileus between succus entericus reinfusion (SER) group and the control group. Time to first passage of flatus or stool after surgery in the SER group (27.9 ± 6.02 h) is significantly shorter than the control group (32.3 ± 6.26, hours *p* = 0.004). Compared with the control group (5.52 (4.0–7.0) days), postoperative length of stay in the SER group was 4.90 (3.0–7.0)days (*p* = 0.009). As for low anterior resection score(LARS), the SER group had a lower score 1 week after discharge than the control group (*p* = 0.034). However, 1 month after discharge, the LARS in the two groups had no significant difference.

**Conclusions:**

Self-administered succus entericus reinfusion is a feasible prehabilitation management for outpatients and can improve better outcomes. Compared with non-reinfusion group, succus enterius reinfusion group displays significantly shorter time for gastrointestinal function recovery and postoperative hospital stay without increasing complication, and it can bring better quality of life in a short term.

## Introduction

Rectal cancer is one of the most common carcinomas worldwide [1]. Prophylactic ostomy is widely applied to reduce the morbidity and mortality associated with anastomotic complications after proctectomy, especially for patients with low rectal carcinoma or neoadjuvant therapy. Both ileostomy and transverse colostomy are effective for fecal diversion, however, loop ileostomy was associated with less parastomal complications and improved quality of life [2]. Loop ileostomy is widely accepted due to its easy creation and closure [3].

Postoperative ileus(POI) with the reported incidence varies from 8.0 to 32% [4, 5] is the most common complication after loop ileostomy closure. Resulting from nociceptors stimulation by direct intestine manipulation and muscular layer infiltration by inflammatory cytokines, delayed recovery of gastrointestinal function can last up to 5 days after surgery, which leads to increased perioperative morbidity and health care costs [6, 7].

After ileostomy closure surgery, patients may have diarrhea, urgency, or incontinence due to long-term exclusion of the distal colon or surgical damage. Low anterior resection syndrome (LARS) refers to a combination of symptoms consist of incontinence for stool and/or feces, increased frequency of bowel movements, and urgency after low anterior resection for rectal cancer [8, 9]. With a reported incidence of up to 60%, LARS has a detrimental impact on patients’ quality of life [10].

Previous studies have demonstrated that ileostomy results in intestinal villi atrophy and loss of segmental contractility in the efferent limb, which may contribute to delayed recovery of motor function after ileostomy closure [11, 12]. To improve absorptive and motor function of the efferent limb before stoma closure surgery, various efforts have been attempted to reestablish intestinal continuity by chyme or thickening agent reinfusion [13, 14].

**Fig. 1:**
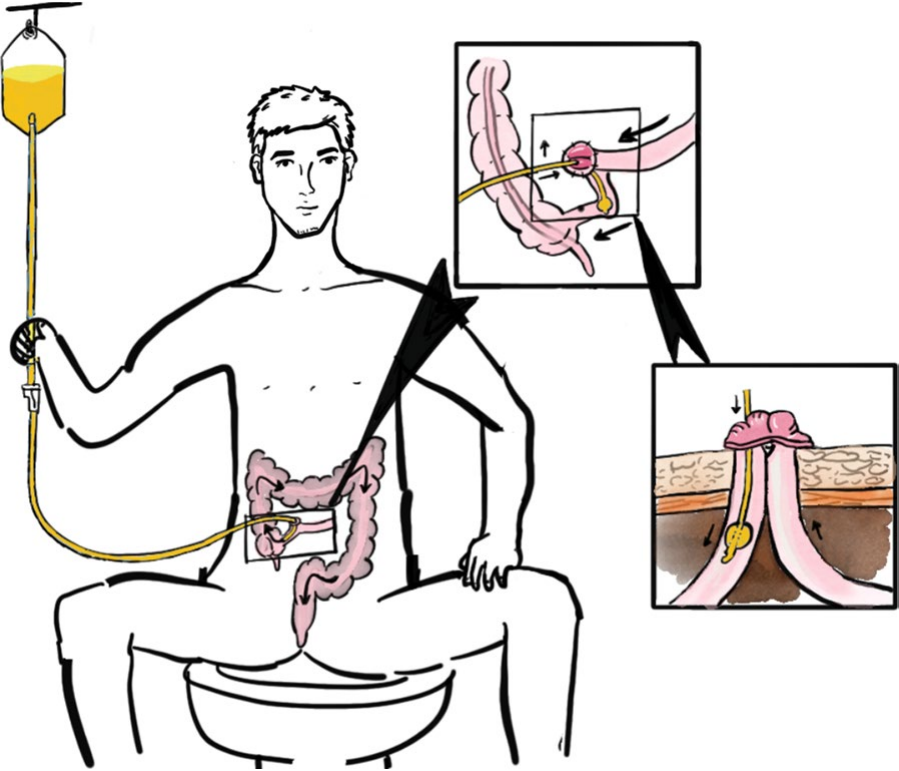
Illustration of self-administered succus entericus reinfusion in out-patient setting

Succus entericus reinfusion (SER) is a method that reinfuses the intestinal fluid collected from the afferent limb into the efferent limb, see Fig. 1. When used in intestinal fistula, SER shows promising results, which may be due to restoration of gut microbiota, absorption and motility [15, 16]. However, whether SER can improve postoperative outcomes after ileostomy closure remains unclarified.

The main objective of this retrospective study is to evaluate self-administered succus entericus reinfusion daily through efferent limb of loop ileostomy 2–4 weeks before closure can improve short-term outcomes.

## Materials and methods

### Patients

This study is a retrospective analysis based on prospectively collected data. All patients received index surgery (low anterior resection) for rectal cancer and protective loop ileostomy during the operation, and they underwent ileostomy closure later. The including criteria are age 18–80, irrespective of receiving neo-adjuvant therapy or adjuvant therapy; ASA class I to II; survived at least 2 months after stoma closure surgery. Patients received more than one intestinal operations (n = 5), with missing data (n = 11), unable to provide informed consent due to various reasons (n = 4) were excluded. Patients (n = 3) who were unable to finish the reinfusion daily without the supervision of health care staff were excluded.

From May 2016 to November 2019, a total of 134 patients underwent ileostomy closure in our hospital; and 72 patients meet the included criteria and were enrolled for this research. A total of 30 patients with SER (SER group) and 42 patients without SER (non-SER group) were analyzed in this study. All participants received colonoscopy and meglumin diatrizoate enema before ileostomy closure surgery to exclude the presence of anastomotic leakage or stricture, which is routine examination before surgery. Colonoscopy Imaging was taken and compared in patients with or without SER.

All patients were informed of the protocol of this study and signed the informed consent prior to inclusion in this study. This study will be conducted in line with the declaration of Helsinki. The study protocol was approved by the ethical committee of the Affiliated Hospital of Qingdao University (QYFY WZLL 25860).

### Self-administered succus entericus reinfusion

Patients who had a better treatment compliance and can perform the succus entericus reinfusion in home properly were grouped into the SER group. Patients were trained on reinfusion of succus enetricus in a follow-up visit before admission(see Fig. 1). SER was done daily in an outpatient setting 2–4 weeks before stoma closure surgery with the insertion of a 16^#^ or 18^#^ Foley catheter into the efferent limb after sufficiently lubricated (Patent No. ZL 201720668812.2). The fresh succus entericus collected from the afferent limb or ostomy bag was filtered to remove the large impurity and diluted by 500–1000 ml normal saline depended patients’ weight. No side effects such as efferent loop damage or abdominal pain was observed throughout the course of treatment. No patient received medications affecting bowel movements before stoma closure surgery.

### Ileostomy closure

All patients received the same following protocol. Antibiotics prophylaxis with 3rd generation cephalosporin was administered 30 min before skin incision. The reversed side-to-side type (π) was adapted for ileal-ileal anastomosis using 60 mm linear stapler. The abdominal wall and skin were closed with interrupted absorbable and monofilaments sutures separately. All the operations were under general anesthesia without spinal anesthesia. Both the surgeons and anesthesiologists were blinded to the patients’ SER status.

### Postoperative protocol

The surgical team which performed the ileostomy closure surgery managed the patients during the postoperative period. Oral clear fluid intake was allowed the next day after surgery provided that the patient didn’t have abdominal pain, nausea, or vomiting. The frequency was gradually increased with patient’s good tolerance to clear liquids. Ambulation was encouraged at the same time. After passage of stool or flatus, liquid diet or soft food was provided. Postoperative diarrhea was defined as more than 3 times of diarrhea with 24 h.

The primary outcome is postoperative ileus(POI). POI was defined as inability to tolerate oral diet or absence of flatus over 72 h, with no evidence of mechanical obstruction either clinically or radiologically [17, 18].

The secondary outcomes are time to first passage of flatus or stool, postoperative length of stay. The criteria for discharge were oral diet tolerance, passage of stool or flatus, absence of complications (fever, surgical site infection) and ambulation.

Follow-up visits were scheduled 1 week and 1 month after discharge. The LARS score was appraised during these two visits separately.

### Statistical analysis

Continuous variables are expressed as means and standard deviation (SD). Categorical data are expressed as frequency and percentage.The data was analyzed by BM SPSS Statistics 25 for Mac (SPSS 25 Mac). The differences between SER group and non-SER group were analyzed by Chi-square test for categorical data and Student’s-t test for continuous data. p values of < 0.05 were considered statistically significant.

## Results

The demographic characteristics of SER and non-SER group of patients are shown in Table 1. There is no significant difference regarding age, gender, ASA classification, BMI, distance from tumor to anal edge, number of patients receiving neoadjuvant therapy, surgical approach, number of patients receiving neoadjuvant and adjuvant therapy.

**Table 1:** Demographic characteristics of SER and non-SER group of patients

	SER group (n = 30)	Non-SER group (n = 42)	p value
Age, year, median (range)	64(46–77)	65(41–78)	0.661
Gender (male/female)	14/16	18/24	0.812
ASA classification			0.447
I	8	16	
II	22	26	
Body mass index at index surgery (kg/m^2^)	25.46 ± 1.98	26.49 ± 2.95	0.101
Body mass index at stoma closure (kg/m^2^)	24.40 ± 1.96	25.23 ± 2.62	0.151
Distance from tumor to anal edge(cm)	9.10 ± 3.48	8.14 ± 2.09	0.151
Neoadjuvant therapy	6/24	10/32	0.780
Surgical approach of index surgery: laparoscopic/open	28/2	38/4	1.000
Inter-surgery period (days)	210.2 (98.0–413.0)	150.7 (43.0–347.0)	0.046
Albumin prior to stoma closure (g/l)	42.34 ± 4.15	42.61 ± 5.98	0.830
WBC (10^9^/l)	5.51 ± 1.49	4.9161 ± 1.42	0.089
RBC (10^12^/l)	4.17 ± 0.27	4.27 ± 0.30	0.160
Lymphocyte (10^9^/l)	1.43 ± 0.54	1.26 ± 0.62	0.228
Adjuvant therapy	16/14	20/22	0.811

We also analyzed postoperative outcomes between groups (Table 2). Compared with the control group (32.3 ± 6.26 h), the time to first passage of flatus or stool in the SER group was 27.9 ± 6.02 h (*p* = 0.004). In the SER group, a slightly longer inter-surgery period was observed than in the control group, *p* = 0.046. There was no significant difference in the incidence of postoperative ileus between succus entericus reinfusion (SER) group and the control group.

**Table 2:** Postoperative outcomes of SER and non-SER group

	SER group (n = 30)	Non-SER group (n = 42)	p value
Operation time (min)	54.851 ± 13.1	51.551 ± 11.8	0.217
Blood loss (ml)	14.5 ± 8.3	12.7 ± 8.5	0.385
Time to first passage of flatus or stool (h)	27.9 ± 6.02	32.3 ± 6.26	0.004
Postoperative ileus	3/27	4/38	1.000
Postoperative diarrhea	3/27	13/29	0.046
Other complications	4/27	7/35	0.506
Albumin prior to discharge (g/l)	36.1 ± 2.04	36.54 ± 3.19	0.521
Postoperative Length of stay (days)	4.90 (3.0–7.0)	5.52 (4.0–7.0)	0.009

The incidence of postoperative ileus in the succus entericus reinfusion (SER) group was 10%, no significant difference was found when compared with the control group (9.5%). Additionally, postoperative length of stay in the SER group (4.90 (3.0–7.0) days) is significantly shorter than the control group (5.52 (4.0–7.0), *p* = 0.009).

Table 3 shows the low anterior resection score (LARS), the SER group had a much lower LARS 1 week after discharge than the control group, p = 0.034. However, 1 month after discharge, the LARS in the two groups had no significant difference.

**Table 3:** The LARS in SER and non-SER group after discharge

	SER group (n = 30)	Non-SER group (n = 42)	p value
1 week after discharge			0.034
No LARS (0–20)	14	11	
Minor LARS (21–29)	11	12	
Major LARS (30–42)	5	19	
1 month after discharge			0.220
No LARS (0–20)	13	14	
Minor LARS (21–29)	13	15	
Major LARS (30–42)	4	13	

**Fig. 2:**
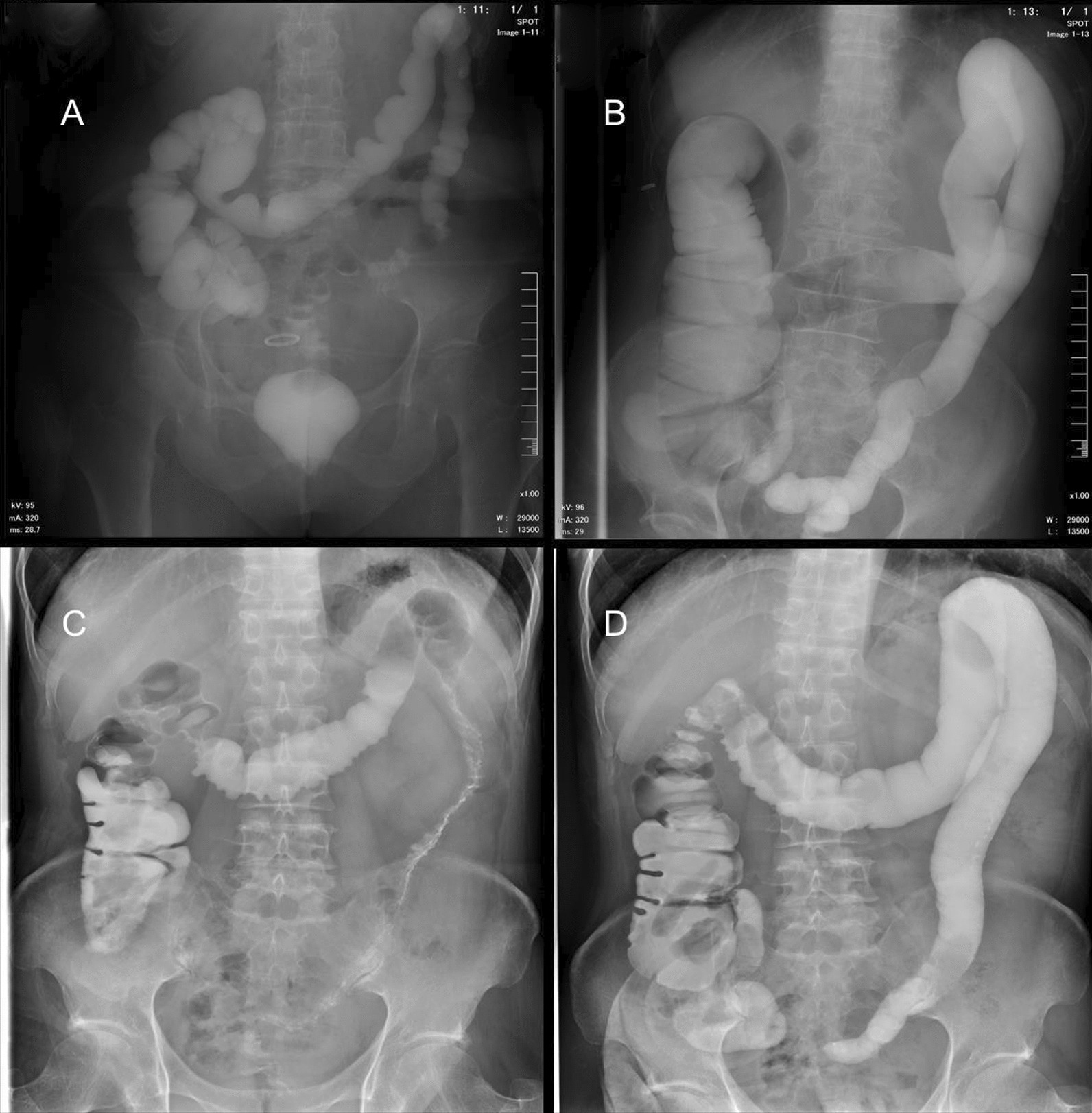
Abdominal Imaging in patients with or without succus entericus reinfusion. **A** A patient without succus entericus reinfusion. **B** Another patient 2 weeks after succus entericus. **C** The patient prior to succus entericus reinfusion. **D** The same patient with C 2 weeks after succus entericus reinfusion

**Fig. 3:**
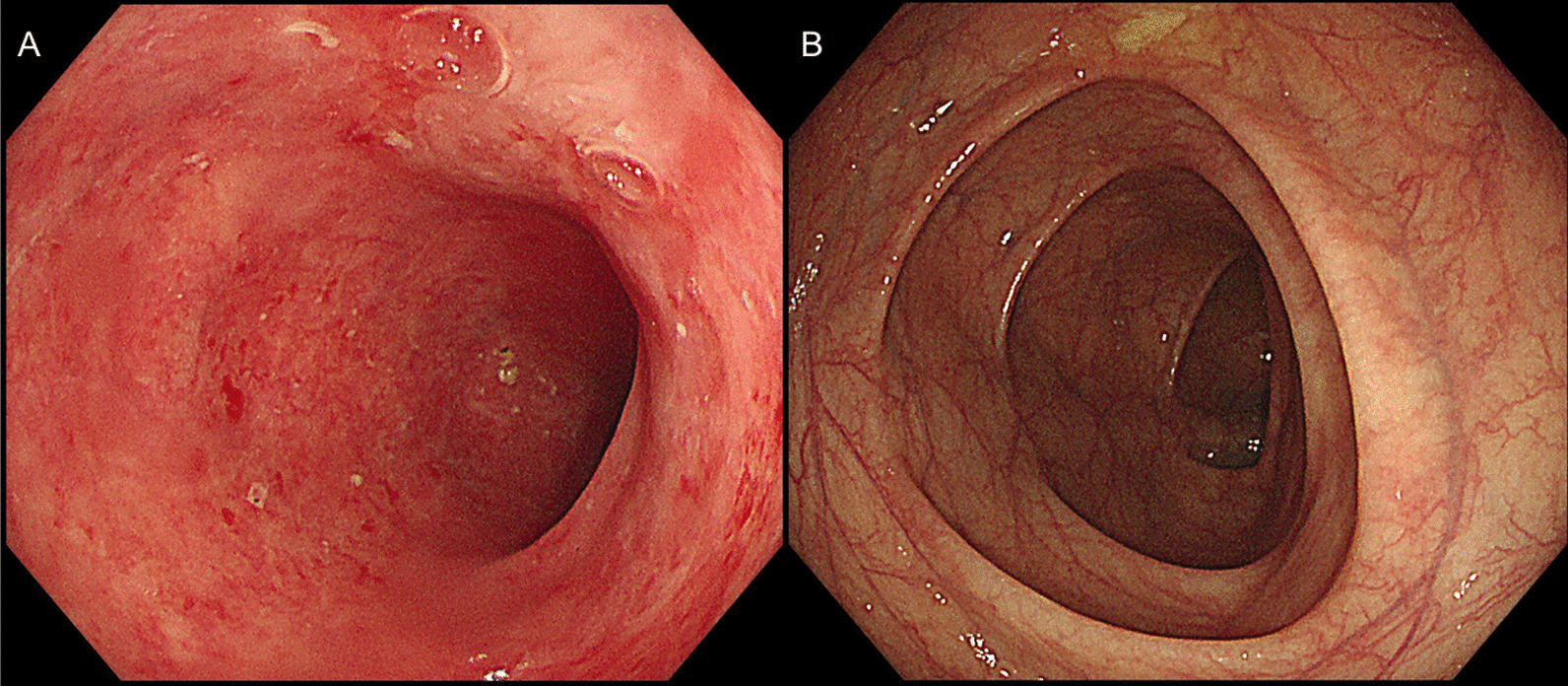
Colonoscopy Imaging(50 cm distant from anal edge) in patients after prophylactic ileostomy with or without succus entericus reinfusion. **A** Inflammation and edema of the defunctionalized, bypassed colon after ileostomy. **B** Normalization of mucosa in the bypassed colon 2 weeks after succus entericus reinfusion

Figure 2A shows the collapsed efferent limb in the patient without succus entericus reinfusion. Recanalization of the efferent limb was observed after 2-week reinfusion in another patient (Fig. 2B). Figure 2C and D display the abdominal imaging in the same patient prior to and after succus entericus reinfusion separately. The collapsed limb shown in Fig. 2C was recanalized 2 weeks after succus entericus reinfusion, as shown in Fig. 2D. Prior to ileostomy closure surgery, the colonic mucosa showed inflammation, edema, and friable, see Fig. 3A. Two weeks after succus entericus reinfusion, colonic mucosa returned to normal under colonoscopy as seen in Fig. 3B. Although there was’t certain quantified index to evaluate the image and endoscope changes of SER, significant improvement was observed.

## Discussion

Protective ileostomy may be performed under elective or emergency situations, for benign or malignant diseases, as permanent or temporary [2]. By diverting fecal stream and minimizing clinical consequences of anastomotic leakage, defunctioning ileostomy was often performed during low anterior resection for rectal cancer in patients with increased risk of anastomotic dehiscence, such as advanced age, poor controlled diabetes, neoadjuvant therapy prior to stoma closure surgery, long-term use of glucocorticoids or immunosuppressive medications [19]. However, ileostomy itself is not devoid of complications. Readmissions, dehydration, prolapse, renal failure, etc. have been reported [20, 21]. Furthermore, ranging from 3 to 40% [5, 22], complications related to loop ileostomy reversal have been demonstrated in different series, such as surgical site infections, postoperative ileus, diarrhea or constipation, or even mortality [23].

POI is the most common complication after ileostomy reversal surgery [24]. In a recent systematic review and meta-analysis conducted by Richard Garfinkle [4], the pooled estimate of POI was 8% (range from 6.7 to 12.4% varied by definition). The incidence of POI in our study is 9.7%, which is in accordance with Richard’s study and many previous series [25, 26]. Aside from lengthen time of stay, delayed recovery of gastrointestinal function is related with increased healthcare costs and risk for other morbidities [27]. Hence, efforts to improve gastrointestinal function after stoma closure should be made to minimizing the burden both economically and clinically.

After a stoma has been introduced, a variety of structural and functional changes took place in the efferent limb of ileostomy due to its’ exclusion of bowel transit. In the bypassed segment of intestine, villi atrophy, muscular layer weakness, gut microbiota imbalance, and endocrine disturbance have been revealed by previous studies [11, 28, 29]. In our study, the colonic mucosa showed inflammation, edema, and friable prior to ileostomy closure surgery, see Fig. 3A. All the pathophysiologic processes above have separately or in jointly contribute to insufficient intestinal absorption and decreased motility, which finally result in impaired gastrointestinal function recovery or changed bowel habits after restoration of intestinal continuity. To better prepare the intestine before stoma closure, Abrisqueta [14] and his colleagues stimulate the efferent limb via a solution consist of normal saline and thickening agent. As a result, the time to first flatus or stool was significantly shorter in the simulated group. Studies using chyme reinfusion into the efferent limb of temporary ileostomy in Crohn’s patients also showed desirable results [13]. According to H R Rosen’s study, prophylactic transanal irrigation has been shown to prevent symptoms of LARS for up to 3 months [30]. In our study, patients with better treatment compliance can reinfuse succus entericus in an outpatient setting. Normalization of mucosa was seen in the bypassed colon 2 weeks after SER, see Fig. 3B. However, there are some potential risks for those with poor compliance. The insertion of Foley catheter into the efferent limb may damage the intestinal mucusa or intestinal wall.

Without reference to the impact of altered gut microbiota in efferent limb after the introduction of ileostomy, previous studies only put an emphasis on the stimulation or nutrition support of efferent limb prior to stoma closure [14, 31, 32]. Succus entericus reinfusion can maintain the structure and function of intestinal mucosa, protecting the intestinal mucosal barrier. Adaptive regulation takes place immediately after ileostomy has been introduced. The mucosa of afferent limb displayed hypertrophy in short-term as evidenced by increased expression of peptide YY content [29]. With the overexpression of nNOS, the lumen of afferent limb in animal models dilated shortly in response to morphological changes in the myenteric network [33]. On the contrary, the mucosa of efferent nonfunctioning limb tends to be atrophic and fibrotic, which is adaptive to the deprivation of nutrition and bowel movements. Diversion colitis refers to an inflammatory disorder in bypass colon segments that are diverted from fecal stream after ostomy surgery [34, 35]. It is hypothesized that the lack of luminal nutrients in colonocytes and imbalance of gut microbiota may contribute to the development of diversion colitis [36]. Our study shows that the reinfusion of succus enterisus can decrease inflammation of the bypassed colon, however, the underlying mechanism need to be clarified.

Manifested by relatively reduced Candidate Genera (notably Clostridia and Streptococcus), the microflora within the defunctioned efferent limb is less diverse [37]. It is reasonable to assume that dysbiosis in response to ileostomy-related nutrient deprivation and impaired mucosal renewal in the efferent limb contribute to the deterioration of host-microbial interactions, and finally postoperative complications after stoma closure. We hypothesize that strategies to maintain microflora homeostasis prior to reanastomosis could improve postoperative outcomes after ileostomy reversal surgery. The succus entericus contained nutrients and balanced microflora from the afferent limb was reinfused freshly into the efferent limb with saline dilution. The decreasing amount of stool eliminated from the anus indicates that the efferent limb is better prepared for reanastomosis after succus entericus reinfusion. Our study reveals that SER after ileostomy is associated with shorter length of stay following stoma reversal procedure. Further research may focus on the changes of mucosal structure and microflora profiles in the reinfused intestine.

There are some potential limitations in this study. First, the retrospective non-randomized nature and small sample size of each group may undermine the ability to detect trivial but clinically significant differences, which can lead to Type II error. We have registered prospective trials to further evaluate the effect of succus entericus on postoperative outcomes after stoma reversal surgery. Second, during preadmission visits, patients in the SER group showed high compliance with this technique because self-administered reinfusion is convenient, economical, and easy to perform in an outpatient setting, however, there are some patients who are unable to finish the reinfusion daily without the supervision of health care staff. The exclusion of this group of patients may lead to selection bias. Third, as a single-center study, our results may not be applicable to other institutions. In the future, larger-scale multicenter studies are crucial to validate the results of our current findings.

## Conclusion

For patients with protective ileostomy, self-administered succus entericus reinfusion prior to stoma reversal is an economical and feasible strategy and can be performed in outpatient settings. When performed daily for a period of 2–4 weeks, this method can improve postoperative outcomes. The time to first passage of flatus or stool and the length of postoperative stay are significantly shortened compared to the non-reinfusion group. Evidenced by LARS, the short-term quality of life (1 week after discharge) was more desirable in the SER group than the control group. Further research needs to be conducted to clarify the mechanisms underlying the beneficial effect of succus enterisus on the function of the bypassed intestine segment.

## Data Availability

The datasets used and/or analysed during the current study available from the corresponding author on reasonable request.
