# Correction to: Self-administered succus entericus reinfusion before ileostomy closure improves short-term outcomes

**DOI:** 10.1186/s12893-023-02195-0

**Published:** 2023-09-19

**Authors:** Zhen Liu, Liang Fang, Liang Lv, Zhaojian Niu, Litao Hou, Dong Chen, Yanbing Zhou, Dong Guo

**Affiliations:** 1 Department of Emergency Surgery, The Afliated Hospital of Qingdao University, Qingdao, Shandong China; 2 Department of Gastrointestinal Surgery, The Afliated Hospital of Qingdao University, No.16 Jiangsu Rd, Qingdao, 266000 Qingdao, Shandong China; 3 Department of Gastroenterology, The Afliated Hospital of Qingdao University, Qingdao, Shandong China; 4 Department of General Surgery, The Afliated Hospital of Qingdao University, Qingdao, Shandong China

Following publication of the original article [1], in this article the Funding information section was missing from this article and should have read “Project ZR2020QH037 supported by Shandong Provincial Natural Science Foundation”.

The original article has been corrected.

## References

[1] Liu Z, Fang L, Lv L, et al. Self-administered succus entericus reinfusion before ileostomy closure improves short-term outcomes. BMC Surg. 2021;21:440. 10.1186/s12893-021-01444-4. 10.1186/s12893-021-01444-4 PMC8713408 34961502

